# Diagnosing acute compartment syndrome—where have we got to?

**DOI:** 10.1007/s00264-019-04386-y

**Published:** 2019-08-29

**Authors:** Tristan E. McMillan, William Timothy Gardner, Andrew H. Schmidt, Alan J. Johnstone

**Affiliations:** 1 Department of Trauma & Orthopaedics, Aberdeen Royal Infirmary, Foresterhill, Aberdeen, AB25 2ZN UK; 2 Department of Orthopaedic Surgery, Hennepin County Medical Center, 701 Park Avenue South, Mail Code G2, Minneapolis, MN 55415 USA

**Keywords:** Compartment syndrome, Ischaemia, Trauma, Investigation, Diagnosis

## Abstract

**Purpose:**

Acute compartment syndrome is a condition whereby tissue ischaemia occurs due to increased pressure in a closed myofascial compartment. It is a surgical emergency, with rapid recognition and treatment—the keys to good outcomes.

**Methods:**

The available literature on diagnostic aids was reviewed by one of the senior authors 15 years ago. Now, we have further reviewed the literature, to aim to ascertain what progress has been made.

**Results:**

In this review, we present the evidence around a variety of available diagnostic options when investigating a potential case of acute compartment syndrome, including those looking at pressure changes, localised oxygenation, perfusion, metabolic changes and available blood serum biomarkers.

**Conclusions:**

A significant amount of work has been put into developing modalities of diagnosis for acute compartment syndrome in the last 15 years. There is a lot of promising outcomes being reported; however, there is yet to be any conclusive evidence to suggest that they should be used over intracompartmental pressure measurement, which remains the gold standard. However, clinicians should be cognizant that compartment pressure monitoring lacks diagnostic specificity, and could lead to unnecessary fasciotomy when used as the sole criterion for diagnosis. Therefore, pressure monitoring is ideally used in situations where clinical suspicion is raised.

## Introduction

Despite widespread awareness amongst clinicians, acute compartment syndrome (ACS) remains a devastating condition to patients in which it is diagnosed. It is a surgical emergency, with prompt identification and early implementation of management being key to a more favourable outcome. In 2003, one of the senior authors reviewed and summarised the literature, focussing on the early identification and diagnosis of acute compartment syndrome [1]. Now, 15 years later, we have performed a further review of the literature to provide an update on the most current evidence.

ACS represents tissue ischaemia that is associated with an increase in pressure within a closed myofascial compartment [2]. It most commonly occurs in the lower leg [3] but can occur within any myofascial compartment in the body, including the upper limb [4, 5], thigh [6], abdomen [7] and buttocks [8]. The commonest cause of ACS is trauma; 40% of all trauma-related acute compartment syndromes occurring after fractures of the tibial shaft, and the incidence in this injury group is around 1 to 10% [9–12]. ACS following high-energy injuries of the tibial plateau is often overlooked, despite the incidence being at least as high as in displaced fractures of the tibial shaft [13]. A further 23% occur solely as a result of soft tissue injuries, and fractures of the forearm account for a further 18% [14]. ACS is more common in young people [15], and therefore, in addition to significant morbidity, it also has social and financial impacts should it occur.

There are other causes for ACS in addition to bony trauma, and it can occur anywhere in the body. Examples include static patient positioning during long surgical procedures, especially those associated with the Lloyd-Davies position in combination with Trendelenburg tilt [16–20], and the injection of recreational drugs [21]. Specific to the lower limb, a number of other causes are reported in the literature. Simply placing the limb in a cast can increase intracompartmental pressure, and subsequent bi-valving of a cast can reduce intracompartmental pressure by as much as 47% [22]. ACS has also been seen following lower limb arthroplasty, most commonly as a result of prolonged static positioning, post-operative bleeding and possible tourniquet use [23]. Additionally, injury to blood vessels such as rupture of the superior gluteal artery [24, 25], rhabdomyolysis [26] and sports injuries in the absence of fractures [6] can all lead to ACS.

### Pathophysiology

The true pathophysiology of compartment syndrome remains a contentious issue that is debated amongst researchers. The most popular hypothesis is the arteriovenous pressure gradient theory, which states that when intracompartmental pressure rises due to a traumatic event, venous pressure also rises as a result, effectively damming blood flow through the injured limb. This leads to a decrease in the arteriovenous pressure gradient, and therefore the blood flow to the tissues is compromised, eventually resulting in tissue ischaemia [27]. The rise in venous pressure itself further increases the intracompartmental pressure due to increased tissue oedema. As tissue ischaemia occurs, cell lysis releases intracellular components into the interstitial fluid which cause further fluid accumulation by osmosis [3]. Reversible neuropraxia due to ischaemia can occur within 1 h and evidence of muscle necrosis can be seen after as little as 3 h [28, 29], so prompt diagnosis is crucial to avoid devastating consequences in this often-young patient cohort.

### Clinical examination

Clinical suspicion supplemented by careful, repeated clinical examination continues to be the clinician’s greatest tool in the diagnosis of ACS. Traditional teaching emphasises ‘pain out of proportion to the injury’ as a cardinal symptom which, together with the patient’s mechanism of injury, should increase the index of suspicion [30]. Additionally, exacerbation of pain on passive stretching of the muscles within the involved compartment has a specificity of 97% and negative predictive value of 98%, but only a sensitivity of 19% and positive predictive value of 14% [31]. Pain is subjective, making the diagnosis more difficult. In the late stages, pain can be diminished due to paraesthesia/anaesthesia resulting from nerve ischaemia and there are some rare cases of ACS presenting in the absence of pain [32, 33]. In these cases, a more thorough assessment of signs, symptoms and clinical examination is essential to guide decision-making. The remaining 4 “P’s” (pallor, pulselessness, paralysis and paraesthesia) of ACS are mostly late signs after prolonged ischaemia and subsequent significant neurovascular injury [16]. Additionally, they can lack specificity and sensitivity—their presence is often seen in association with other pathology [3], especially chronic limb ischaemia due to arterial disease [30].

A particularly challenging group in which to diagnose ACS are those who have impaired consciousness, especially those who are intubated and ventilated. These patients are unable to alert clinicians to the early symptoms of ACS, and although clinical examination has its place, quantitative investigations play a vital role in monitoring and prompt detection of ACS.

### Clinical investigations

#### Compartment pressure monitoring

Compartment pressure monitoring attempts to objectively assess the pressure within each muscle compartment, but there is controversy around its routine use [34–36], especially the value of pressure data compared with regular clinical review, and threshold pressures at which to intervene surgically [3].

A variety of pressure monitoring equipment is available, including traditional needle manometry (Whiteside’s’ technique) [34], transducer and multi-parameter monitors usually used to monitor arterial blood pressure [37] and dedicated compartment pressure monitors (e.g. transducer tipped intracompartmental pressure monitors). Pressure measurements obtained with any of these devices are significantly more sensitive and specific for diagnosing ACS than clinical examination alone. With such diagnostic accuracy, we must question why pressure monitoring is not used routinely in assessing injured patients for ACS, but it is mostly as a consequence of concern that pressure monitoring, regardless of whether it is done as a single measurement or with continuous measurement, may lead to overtreatment [38, 39]. Significant interobserver variability in measurements has also been reported [40].

Over the years, the threshold pressure at which a surgeon should intervene has been widely experimented and remains unresolved. The most common threshold used by clinicians when assessing compartment pressures remains the delta pressure (ΔP), which is the diastolic blood pressure minus the intracompartmental pressure. Δ*P* less than or equal to 30 mmHg is defined as ACS [16, 30, 35, 38, 41]. However, it has been suggested that compartment pressures should be used to confirm clinical suspicion, rather than as a screening tool in the entire population. Garner et al. [38] reviewed a number of studies where patients had a Δ*P* ≤ 30 mmHg, but were asymptomatic, and so strict use of this cut-off would have resulted in a number of unnecessary operations. One suggestion to improve the accuracy of measurements is that the pressure should be recorded in a zone within 5 cm of any fracture but not communicating with the fracture itself [30].

Recent research suggests that the trend in compartment pressure over time is much more useful than a single pressure, as the latter approach is associated with a significant (35%) false-positive rate [39]. Additionally, McQueen et al. found the sensitivity and specificity of continuous intracompartmental pressure monitoring to be high, with estimated positive and negative predictive values of 93% and 99% respectively [35]. The proposed benefit of continuous monitoring is that it reduces the influence of potentially erroneous single readings, but additionally it allows a trend to be identified that may well facilitate the diagnosis prior to clinical signs developing. More research is required to refine the potential benefits of continuous pressure monitoring and what the most accurate pressure threshold is.

**Fig. 1:**
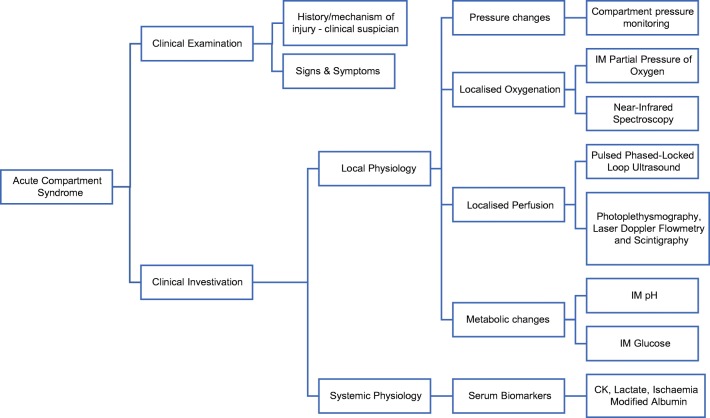
Acute compartment syndrome: currently available investigations

With continued questions regarding the diagnostic utility of compartmental pressure monitoring, research is ongoing to investigate alternative methods that are less focused on pressure and more on ischaemia. Therefore, the focus is shifting to measuring changes in haemodynamic and metabolic parameters in diagnosing ACS (Fig. 1).

#### Monitoring localised oxygenation

##### Intramuscular partial pressure of oxygen

Measurement of the partial pressure of oxygen has been investigated in canine models where ACS had been induced [42, 43], with promising results. Doro et al. measured the intramuscular partial pressure of oxygen in adult beagles to be 62–65 mmHg before creation of compartment syndrome. They found that a partial pressure of oxygen of less than 30 mmHg had a 100% sensitivity (95% CI 72–100%) and 100% specificity (95% CI 69–100%) [43]. Feasibility studies looking at direct measurement of intramuscular oxygen pressures in humans are available [44, 45], although study numbers are small.

Oxygen saturation can also be monitored non-invasively, as shown by Reisman et al. [46], Shuler et al. [47, 48] and Schmidt et al. [36] in their trials using near-infrared spectroscopy (NIRS) to measure oxygen saturation of muscle.

##### Near-infrared spectroscopy

The principals of measuring blood flow are based on the fact that as a response to trauma, the body will increase the blood flow to the site of injury. If this is absent, it could be indicative of ACS and would also result in desaturated haemoglobin and myoglobin [49]. NIRS is a non-invasive method for assessing levels of oxygenated muscle haemoglobin and myoglobin. When blood flow is decreased, a reduction in local oxygen saturation and muscle oxygen tension can be seen. It is not new technology, having been used over 40 years ago to monitor cerebral and myocardial oxygen sufficiency [50]. This was achieved through transillumination of the heart or brain, with photon transmission through the organ at wavelengths varying from 740 to 860 nm focused to cytochrome aa3. Its potential role in diagnosing ACS is significantly more recent. NIRS was proposed by Reisman et al. [46] as a non-invasive technique for assessing blood flow to rule out ACS. The technology is similar to measuring oxygen saturations in subcutaneous tissues, something that is done routinely in bedside monitoring of patients.

In a porcine model of ACS, near-infrared spectroscopy provided a reliable, sensitive measure of tissue oxygenation that directly correlated with a simulated increase in tibial compartmental pressure and a decrease in tibial intracompartmental perfusion pressure [51]. In 2007, a paediatric case report utilised NIRS in the diagnosis of ACS in a one month old infant, with a NIRS value of 15% in the involved limb versus 40–50% in the uninvolved limb [52]. In patients with ACS, Shuler et al. found NIRS values from at least one compartment to be more than 3% below an uninjured control compartment [53]. They advocated simultaneous monitoring of the same compartment on the contralateral limb in order to provide a control. This was because NIRS values vary significantly between individuals and indeed can vary within compartments over time, but the simultaneous difference between uninjured ‘like’ compartments varies less. Just as for pressure monitoring, continuous assessment of tissue oxygenation with NIRS would theoretically be an ideal means to document the onset or progression of tissue hypoxia, alerting clinicians so that timely intervention follows. However, a study by Schmidt et al. found poor reliability in obtaining continuous NIRS data not only in the injured limb, but even in the control (reference) limb, raising questions about the value of continual NIRS in diagnosing ACS [54]. Therefore, the literature is somewhat confounding. Whilst NIRS has the potential to offer the ability to objectively measure tissue perfusion in a non-invasive and continuous manner, additional studies are required to validate whether or not NIRS has a clinical role in ACS monitoring. Other limitations of NIRS include limited depth of tissue penetration and the tendency for skin colour and subcutaneous bruising/haematomas to adversely affect readings.

#### Monitoring localised perfusion

##### Ultrasound and pulsed phased-locked loop ultrasound

In 1989, ultrasound was used to assess for variations in compartmental geometry and echogenicity in relation to pressure, and whilst echogenicity increased as pressure increased, correlation and reproducibility were felt to be poor [55]. More recently, ultrasound has been used to assess compartment elasticity when subjected to external compression, using a standardised pressure. So far it has only been assessed in vitro [56] and in cadaveric models [57] by Sellei et al.

The evolution of pulsed phased-locked loop (PPLL) ultrasound shows greater promise. For the diagnosis of ACS, PPLL measures fascial wall displacement in correlation with arterial pulsations, with the magnitude of arterial pulsations and subsequent perfusion diminishing as compartmental pressure rises. In a cadaveric study, Lynch et al. showed PPLL to detect compartmental changes resulting from pressure changes of 1 mmHg [58]. Wiemann et al. collected data from nine  patients with simulated compartment tamponade and three  patients with ACS and demonstrated a linear correlation between PPLL and intramuscular pressure [59]. Similarly, in a simulated ACS study, Lee et al. showed a statistically significant correlation between PPLL data and increasing chamber/intramuscular pressure [49]. Interestingly, they also found PPLL to perform superiorly to NIRS, due to less subject to subject variability. Having said this, the data in the literature is from small studies performed in a predominantly non-clinical simulated environment, and so considerable research is required before this method could be used clinically.

##### Photoplethysmography, laser Doppler flowmetry and scintigraphy

Photoplethysmography has also been trialled as a way of measuring increasing blood flow by Challa et al. This technique uses a light-emitting diode to shine light onto skin, and a photodetector can monitor and record the attenuation of the reflected light, which is directly related to blood flow [60]. There is no current role to support its clinical use in the diagnosis of ACS. Laser Doppler flowmetry and scintigraphy also provide methods for assessing local perfusion. In the diagnosis of chronic compartment syndrome, both techniques have been found to be viable [61, 62]. In scintigraphy, time-consuming scans, the lack of specificity in a traumatised limb and the inability to perform repeated/continuous examinations make it unsuitable for diagnosing ACS. The use of laser Doppler flowmetry in diagnosing ACS is yet to be evaluated.

#### Localised metabolic analysis

##### Intramuscular glucose monitoring

Doro et al. measured intramuscular glucose in 12 canine models (baseline of 158 mg/dL in control group) and found that an intramuscular glucose concentration of less than 97 mg/dL was 100% sensitive (95% CI 73–100%) and 75% specific (95% CI 40–94%), and that changes could be detected as soon as 15 minutes  after induction of compartment syndrome, when compared against a control [43]. Being able to detect changes, this quickly has huge potential; however, the authors acknowledge that the technology used in this study would need further development of glucose sensors in order for this research project to progress to human trials.

##### Intramuscular pH monitoring

The surface pH of skeletal muscle has been confirmed to be a sensitive indicator of peripheral muscle blood flow [63]. Several methods of recording muscle pH have been used in research. Initially, it was only possible to accurately record this through muscle biopsy, making sampling and analysis time-consuming and repeat sampling unsuitable. The development of probes to measure intramuscular (IM) pH is a more recent concept. In research performed by two of the authors (AJJ and KE), a 1.8-mm glass catheter was connected to an ambulatory pH recorder to measure IM pH. In an initial study of 62 patients, its sensitivity and specificity were found to be significantly higher than intracompartmental pressure monitoring [64]. Invasive pH monitoring continues to be investigated by the senior author (AJJ), with early results indicating that IM pH monitoring has the potential to deliver a more accurate and faster method for diagnosing ACS. The outcome of a multi-centre clinical trial due to commence soon will hopefully provide further validity for this technique.

#### Systemic physiology

##### Serum biomarkers

Following trauma, and after the onset of ACS, the inflammatory response leads to a rise in inflammatory biomarkers such as white cell count (WCC), erythrocyte sedimentation rate (ESR) and C-reactive protein (CRP). Additionally, creatine kinase and lactate levels rise, due to muscle breakdown and anaerobic metabolism respectively. Creatinine kinase levels of > 2000 units/L can be a warning sign of compartment syndrome in a sedated and ventilated patient [65]. Similarly, ischaemia-modified albumin (IMA) has been shown to rise with reasonable sensitivity and specificity in the presence of critical limb ischaemia; however, its role in aiding the diagnosis of ACS remains unclear [66]. Whilst the levels of these biomarkers change in association with acute compartment syndrome, at present, no serum marker has been shown to have adequate sensitivity and specificity to quantify the level of skeletal muscle ischaemia, and therefore cannot be accurately used to diagnosis acute compartment syndrome. However, the testing of regional or local biomarker levels such as intramuscular glucose and pH does offer greater potential.

### Treatment

Whatever method of diagnosis is used to identify ACS, implementation of treatment is time-critical. Simple measures such as splitting casts or unwinding compression dressings can make a huge difference and thus raise the importance of regular review [27].

Fasciotomies are unfortunately associated with significant morbidity including the need for further surgery for delayed wound closure, surgical reconstruction with skin grafting or vascularized flaps, cosmetic problems, pain and nerve injury, permanent muscle weakness and chronic venous insufficiency [3]. With evolving diagnostic technologies, hopefully we will soon be in a position to make an earlier diagnosis and thereby create the opportunity to try treatment using non-surgical or less invasive interventions. For fractures of the lower limb, these treatments may include intermittent pressure pumps to help reduce tissue oedema, swelling and intracompartmental pressures [67, 68], tissue ultrafiltration to remove local interstitial fluid [69], hyperbaric oxygen to promote cellular aerobic respiration, the use of free radical scavenging drugs to minimise the extent of tissue damage and the use of membrane stabilising drugs to limit cell death [70].

Not only is the evolution of diagnostic technology vital for the earlier identification and implementation of these potential treatments, it is also crucial to actively monitor their influence during their research and development. Ultimately the overall aim is to advise clinicians of the need and timing for fasciotomy, should the other non-surgical measures fail to halt or reverse the progression of ACS.

## Conclusion

Acute compartment syndrome is a very serious and potentially limb and life-threatening condition. Clinical suspicion should be high, disproportionate pain and pain on passive muscle stretching is the most sensitive clinical sign, and prompt fasciotomies can result in a significant decrease in morbidity, even if it does result in a number of false-positive diagnoses.

Despite the extensive research into ACS, there are still significant areas lacking in consensus, especially with respect to its diagnosis. The development of a reliable and accurate objective method for detecting ACS at an early stage would be ground-breaking. Since the previous review performed by the senior author, there has been substantial developments in modalities of diagnosis, both with respect to measuring intracompartmental pressure and a developing focus on the haemodynamic and metabolic changes seen in ACS. Whilst research into some of these techniques show promise, there is yet to be any conclusive evidence to suggest that they outperform intracompartmental pressure measurement. Until more evidence becomes available, measuring ICP remains the current gold standard objective diagnostic method but only when clinical signs are also present. There is still no firm agreement as to whether intracompartmental pressure monitoring should be performed as a matter of routine in high-risk injuries, and indeed what to do about the results they produce. However, there is a trend towards continuous pressure monitoring rather than diagnosis based on single pressure readings.
